# Genetic Interventions for Spinocerebellar Ataxia and Huntington’s Disease: A Qualitative Study of the Patient Perspective

**DOI:** 10.3233/JHD-240026

**Published:** 2024-09-10

**Authors:** Nienke J.H. van Os, Mayke Oosterloo, Brigitte A.B. Essers, Janneke P.C. Grutters, Bart P.C. van de Warrenburg

**Affiliations:** a Department of Neurology, Donders Institute for Brain, Cognition and Behavior, Donders Center for Medical Neuroscience, Radboud University Medical Center, Nijmegen, The Netherlands; b Department of Neurology, Maastricht University Medical Centre+, Maastricht, The Netherlands; c School of Mental Health and Neuroscience, Faculty of Health, Medicine and Life Sciences, Maastricht University, Maastricht, The Netherlands; d Department of Clinical Epidemiology and Medical Technology Assessment, Maastricht University Medical Centre+, Maastricht, The Netherlands; e Department for Health Evidence, Radboud Institute for Health Sciences, Radboud University Medical Center, Nijmegen, The Netherlands

**Keywords:** Genetic interventions, Huntington’s disease, spinocerebellar ataxia, antisense oligonucleotides

## Abstract

**Background:**

For various genetic disorders characterized by expanded cytosine-adenine-guanine (CAG) repeats, such as spinocerebellar ataxia (SCA) subtypes and Huntington’s disease (HD), genetic interventions are currently being tested in different clinical trial phases. The patient’s perspective on such interventions should be included in the further development and implementation of these new treatments.

**Objective:**

To obtain insight into the thoughts and perspectives of individuals with SCA and HD on genetic interventions.

**Methods:**

In this qualitative study, participants were interviewed using semi-structured interview techniques. Topics discussed were possible risks and benefits, and logistic factors such as timing, location and expertise. Data were analyzed using a generic thematic analysis. Responses were coded into superordinate themes.

**Results:**

Ten participants (five with SCA and five with HD) were interviewed. In general, participants seemed to be willing to undergo genetic interventions. Important motives were the lack of alternative disease-modifying treatment options, the hope for slowing down disease progression, and preservation of current quality of life. Before undergoing genetic interventions, participants wished to be further informed. Logistic factors such as mode and frequency of administration, expertise of the healthcare provider, and timing of treatment are of influence in the decision-making process.

**Conclusions:**

This study identified assumptions, motives, and topics that require further attention before these new therapies, if proven effective, can be implemented in clinical practice. The results may help in the design of care pathways for genetic interventions for these and other rare genetic movement disorders.

## INTRODUCTION

Huntington’s disease (HD) and some forms of spinocerebellar ataxia (SCA)— such as types 1, 2, 3, 6, 7, and 17— are autosomal dominant neurodegenerative disorders caused by pathogenic cytosine-adenine-guanine (CAG) repeat expansions in disease-specific genes [1, 2]. For both groups of disorders, no cure is available yet [3, 4]. Progressive immobility, dependency on others, the presence of additional cognitive and psychiatric symptoms, and the hereditary aspect impact on the quality of life [5, 6].

Therapeutic interventions are currently being developed for both conditions and the expectations are high for different forms of genetic interventions. Genetic interventions target the ribonucleic acid (RNA) translation of the CAG repeat expansions, by using antisense oligonucleotides (ASO) or micro-RNA [7, 8]. As these treatments are new, long-term risks are not clear. One of the disadvantages is that most genetic interventions need to be administered by intrathecal or intracerebral injections to overcome the blood-brain barrier.

Trials for HD and some of the SCA’s are in various stages, ranging from preclinical proof of concept studies, which are currently being translated into phase 1/2 trials, up to phase 3 clinical trials [9–13].

The patient’s perspective on genetic interventions should play an important role in the clinical development and implementation in clinical practice. To date, only a limited number of qualitative studies on this subject have been conducted. These studies showed that individuals with HD or ataxia are willing to participate in clinical trials of genetic interventions, in particular pre-symptomatic HD mutation carriers [14, 15]. Furthermore, the studies gave insight into the benefits versus risks analysis in patients. However, the willingness to undergo these treatments outside the context of a clinical trial was not explored in these studies.

This qualitative study aims to get more insight in the patients’ thoughts, preferences, and concerns regarding genetic interventions. Since genetic interventions are not available as a regular treatment for SCA and HD yet, hypothetical treatment scenarios were put forward in this study. The results may help healthcare professionals to inform their patients during consultation in the best possible way, and to implement genetic interventions for rare genetic movement disorders in future care.

## MATERIALS AND METHODS

### Participants

This study employed in-depth, semi-structured interviews with persons with a genetically confirmed diagnosis of SCA or HD. Participants were recruited by their neurologists from the Radboud university medical center (BvdW) and Maastricht University Medical Center (MO), the Netherlands. Ten participants were included. All participants gave written informed consent before participation in the study. The study was approved by The Regional Ethics Committee Arnhem-Nijmegen, the Netherlands (file number: 2021-9700).

### Data collection

The following information was collected for each participant: age, gender, diagnosis, age of diagnosis, current functioning level on disease-specific functioning scale [16, 17] and Modified Rankin Scale (MRS) (see Supplementary Material 1), current living situation and highest level of education.

Interviews took place in person, by telephone, or by video conference between December 2021 and January 2022. All interviews were conducted in Dutch by the first author (NvO), lasted 30 to 60 minutes, and were audio-recorded. The interviews were semi-structured, guided by a list of topics and ‘sensitizing concepts’ (see Supplementary Material 2) covering general issues regarding future genetic interventions. The topics were established by a literature search and by conversations with the study team. The literature search identified four papers exploring the opinions of patients with neurodegenerative disorders towards genetic interventions: one paper included patients with ataxia, one with HD, and two papers included patients with Duchenne Muscular Dystrophy [14, 15, 18, 19].

Interviews were conducted until achievement of saturation, meaning no new information emerged from new interviews. A data saturation table is available as Supplementary Material 3.

### Qualitative data analysis

Consistent with standard qualitative research techniques, all semi-structured interviews were based on a topic list (Supplementary Material 2) that was iteratively adapted as the interviews evolved, to ensure the list included all relevant themes.

All interviews were fully transcribed ad verbatim. The transcripts were stored and analyzed with ATLAS.ti (Version 9.1.6, Scientific Software Development GmbH, Berlin, Germany) using a generic thematic analysis. Transcripts were coded one by one by the first author, partly in a deductive manner using the pre-defined topics from the topic list, but also inductively by open coding to allow for new topics.

The next step was axial coding. In this phase the open codes were grouped into categories and connections between these categories were made. The list of categories was used to develop a thematic ‘framework’. Results were summarized in this framework by category and by participant, using spreadsheets in Excel. The framework resulted in the identification of superordinate themes. The themes were discussed in the study team until consensus was obtained.

Quotes were translated in English by the first author (NvO).

## RESULTS

In total, 10 individuals participated in this study, five women and five men, aged between 26 and 60 years (median 47.5 years). Two participants with SCA had SCA1 and three had SCA3. All participants with SCA were symptomatic at the time of the interview; however, one patient with SCA1 had only minor symptoms (participant 1). Five participants had HD, of whom two were premanifest carriers of a pathogenic CAG repeat expansion. See Table 1 for clinical characteristics.

**Table 1:** Clinical characteristics of 10 participants in this study

Participant	Gender	Age	Disease	Manifest	Functioning level ^†^	MRS	Social status	Highest educational qualification ^‡^	Work status	Current therapy
1	M	54	SCA1	–/+	1	0–1	Alone	5	Normal	None
2	F	55	SCA3	+	2	1	Partner, children	4	Normal	None
3	F	46	SCA3	+	2	1	Partner, children	5	Adjusted	Physiotherapy
4	M	60	SCA3	+	2	2	Partner	4	No work	Physiotherapy
5	M	57	SCA1	+	2	1	Partner, children	5	Normal	None
6	F	48	HD	+	11	2	Partner, children	3	No work	Physiotherapy, psychologist, medication (SSRI)
7	M	47	HD	+	6	3	Divorced, children	4	No work	Physiotherapy
8	F	43	HD	+	10	2	Partner	4	No work	Physiotherapy, occupational therapy, psychologist, medication (SSRI)
9	F	29	HD	–	13	0	Alone	5	Normal	None
10	M	26	HD	–	13	0	Alone	5	Normal	None

^†^For the SCA participants, the ‘disease stage of ataxia’ as defined by Klockgether et al. [16] was used (disease stages range from 0 to 4 with the lowest number for the highest functioning level). For the HD participants, the TFC of the UHDRS [17] was used (TFC ranges from 0 to 13 with highest number for the highest functioning level). MRS = Modified Rankin Scale. ^‡^Based on the International Standard Classification of Education (ISCED, 2011).

The qualitative analysis resulted in the identification of the following three superordinate themes (see Table 2): (1) knowledge and assumptions; (2) motives and hopes; and (3) preferences. The themes will be highlighted and described below. Exemplary quotes of the participants are listed in Supplementary Material 4.

**Table 2:** Themes and subthemes

Theme	Subthemes
Knowledge and assumptions	- Background knowledge - Assumptions about benefits - Assumptions about side effects
Motives and hopes	- Willingness to undergo genetic interventions - Goals - Quality of life - Acceptance of (unknown) risks - Burden in families - Motives for trial participation
Preferences	- Patient information - Mode of administration - Expertise, location and costs - Other procedural aspects - Timing - Prioritizing

***Theme 1: Knowledge and assumptions***

*Background knowledge:* Almost all participants had background knowledge about genetic interventions. Participants were aware that genetic interventions target the disease-causing protein and some mentioned that there are two methods to do so. They obtained information from websites and social media of patient associations, and from conversations with their neurologist. Two participants did not have any prior knowledge, but they deliberately decided not to search for this information online because they believed it would negatively influence their wellbeing.

*Assumptions about benefits:* Assumptions about positive effects of putative genetic interventions varied. Some participants believed there would be a high chance of a positive effect, others were not so convinced of positive results. All participants hoped that genetic interventions would stop or slow down progression or delay onset of symptoms. None of them believed that such interventions would be able to cure them. Further, participants hoped that genetic interventions would affect symptoms related to mobility, balance, coordination, and speech (SCA patients), and symptoms related to involuntary movements and cognition (HD patients). None of the HD patients explicitly mentioned behavioral problems.

*Assumptions about side effects:* Overall, participants mentioned they believed the chance severe side effects of genetic interventions would occur, is low. They considered chances between 1% and 25% as ‘low risk’, and chances between 30% and 100% as ‘high risk’. Furthermore, they stated that they thought the risk for a per-procedural bleeding or an infection to be as high as 20% to 40%. Participants who had never had a lumbar puncture in the past, were afraid of possible side effects. The other participants said the side effects of a lumbar puncture would be acceptable. Expected side effects that the participants mentioned are displayed in Table 3.

**Table 3:** Assumptions about possible side effects of genetic interventions

Infection
Pain
Cancer
Depression
Cognitive decline; dementia
Effects that result from changes in protein function
Progression of disease
Rehabilitation after treatment necessary
Temporary effect of treatment
Paresis after intracerebral injection
Spinal cord injury or urinary incontinence after lumbar puncture
Risks associated with general anesthesia for intracerebral injection

***Theme 2: Motives and hopes***

*Willingness to undergo genetic interventions:* Almost all participants immediately said they would undergo genetic interventions. Participants considered a range between 30% to 100% chance of a positive effect to be acceptable.

*Goals:* Prevention of disease progression would be an important motive for the participants to undergo genetic interventions. Most symptomatic participants hoped the treatment would prevent further disease progression, or slow down progression, rather than cure them or improve symptoms. All premanifest HD participants hoped genetic interventions would delay onset of first symptoms, rather than slowing down progression of symptoms once these are present.

*Quality of life:* One of the main motives for patients to try genetic interventions is that they want to maintain an acceptable quality of life. Several participants with SCA mentioned that complete wheelchair dependency would significantly decrease their quality of life.

*Acceptance of (unknown) risks:* Although the willingness to try future genetic interventions was generally high in the participants who were interviewed. Most of them said they would weigh possible risks against benefits. Unknown long-term risks seemed to be less important than known procedural-associated, short-term risks. The risks of all adverse effects that would be acceptable for patients were below 20% to 50%. However, an acceptable risk of severe side effects such as paralysis, more rapid disease progression, or even death, ranged between 1% to 40%. Participants said they would accept higher risks when they would be in more advanced disease stages. One person with SCA noted an acceptable risk of death of 70% if she would be wheelchair bound.

*Burden in families:* There seemed to be an emotional burden of disease in the participants’ families. During all interviews, participants regularly mentioned affected family members and in what way these persons have suffered from the disease, but also how seeing family members suffer did affect their perspectives.

*Motives for trial participation:* Seven out of 10 participants would consider to be enrolled in a trial with genetic interventions. They seemed to be aware of possible higher and unknown risks in case they would participate in a trial, as compared to receiving the treatment as a form of standard care with proven effectiveness. Motives for participants to undergo genetic interventions in trial context were: make a contribution to science, gain knowledge (as a benefit for their children and future generations), the hope for a personal benefit, and an earlier timing of possible treatment. Some participants said they would be more willing to participate in a trial if they were further in their disease course. If they had the choice, they would prefer participation in an earlier trial phase (i.e., safety- and dose-finding trials) over a large placebo-controlled trial, because ‘they would know for sure they would receive the drug and not a placebo’.

***Theme 3: Preferences***

*Patient information:* The majority of participants mentioned they would want more information before the start of genetic interventions, for example about the expected chance of a beneficial effect, the possible side effects and risks, and what procedures the treatment would entail. Most participants preferred to be informed by their neurologist. Two persons did not require more information since they would be willing to participate anyway.

*Mode of administration:* In case they would be given the choice, about half of the participants would prefer an operation with an one-time intracerebral injection over repeated (i.e., several times a year) lumbar punctures. Participants were aware that an intracerebral injection is associated with higher periprocedural risks compared to a lumbar puncture.

*Expertise, location and costs*: Patients seemed to desire that their doctor is familiar with their disease and has a certain level of expertise. Most participants were willing to spend time to travel to a nationwide expert center for genetic interventions, rather than to be treated in the local, nearest hospital by a general neurologist who has no specific expertise on their disorder. However, if participants would need repeated lumbar punctures, they tended to be more open to be treated in a local hospital, as long as there is intensive communication with the doctors in the expert center. Furthermore, continuity of care by one and the same healthcare professional is important for patients. Some said they would be willing to travel abroad for genetic interventions. Other participants had doubts about the quality and costs of treatment in a foreign country.

*Other procedural aspects:* Participants were asked whether other procedures would be of influence on their decision to undergo genetic interventions. For example, what if genetic interventions would require them to travel to the hospital often, undergo regular diagnostic tests or to provide frequent blood or cerebrospinal fluid samples? Most participants said these other procedures would not be of influence.

*Timing:* participants were asked what the ideal timing of genetic treatment would be, without taking the possible adverse effects in consideration. All symptomatic participants said they wanted to be treated as soon as possible. None of them wanted to wait for further disease progression. Some participants stated that being at the end stage of the disease, would be a reason to waive further treatment. Participants were given the hypothetical opportunity to be treated at onset or even in the premanifest stage. All participants said they wished for treatment at onset of symptoms (i.e., early manifest stage) or before onset. The two premanifest HD participants would want treatment before the onset of first symptoms. However, one of them preferred to be treated as late as possible in the premanifest stage (i.e., just before development of first symptoms), to prevent to be confronted with ‘being a patient’ in the premanifest stage.

*Prioritizing:* Participants were asked ‘who should be treated first’ in a scarcity context. Most of them replied that they felt that this would be a personal consideration for each individual, and that first priority should be given to the patients who really want to receive the treatment. Some participants felt that patients in an advanced disease stage should be helped first if the treatment is effective for those. Others believed that early manifest patients would benefit most as they would have a higher quality of life.

## DISCUSSION

This qualitative study explored the perspectives of individuals with SCA and HD towards future genetic interventions.

Most of the participants had background knowledge about genetic interventions. They were realistic and knew genetic therapy is not equal to curation. However, they would accept a high risk of severe side effects. This points out that the willingness to try new treatments is high in HD and SCA patients. However, there seems to be limited understanding and knowledge about possible risks, since the participants’ assumptions were somewhat unrealistic. They mentioned percentages for per-procedural bleeding or an infection to be as high as 20% to 40%. From the literature, we know that these percentages are much lower and follow perhaps 0.3% of lumbar punctures [20]. Patients seem to be unaware of these ‘real’ numbers. It is known that patients in general seem to find it difficult to estimate and interpretate probabilities of risk [21]. As a result, there is a likely possibility that due to the understandably high desire to receive a disease modifying treatment, the risk of side effects are anticipated as negligible.

This corresponds with the outcomes of a recent survey among patients with ataxia [14]. The willingness to participate in a trial with new therapies was high, which was explained by the desire for a cure and lack of approved disease-modifying therapies. However, although common motivations for trial participation were potential benefits, the possible side effects, burden, and costs were reasons for non-participation. Another study among patients with HD also showed that participants‘ desire to participate in molecular therapy trials, independent of study design or therapy goals [15].

It is known, and understandable, that patients with incurable disorders such as SCA and HD are willing and hopeful [15]. However, at this stage, the benefits of genetic interventions are not yet clear as we are still in the trial phase for SCA and HD. A phase 3 trial studying ASO therapy in HD patients was discontinued in March 2021 due to safety concerns. This ‘GENERATION-HD1’ study (clinicaltrials.gov number NCT03761849) was stopped early based on the advice of the Independent Data Monitoring Committee that performed a potential benefit versus potential risk analysis [22]. In addition, the phase 1b/2a ‘PRECISION-HD1 and 2’ studies with ASOs (clinicaltrials.gov numbers NCT04617847 and NCT03225846) showed no significant change in mutant huntingtin protein levels in the intervention group as compared to the placebo group [23]. Although mutant huntingtin protein levels are not as important outcome measures as the clinical effect, we will still need to await further results to get more insight into the possible benefits and risks of genetic treatments.

The motives of participants for undergoing genetic interventions despite high risks, reflect an underlying high emotional burden of disease. In many cases they have to cope with an affected family member suffering from the disease, which is known to have an enormous psychological impact [24]. Also, the fact that their children or future generations may become affected, is a reason to participate in disease-modifying treatment trials. In line with those findings, our results show that having an affected family member or a child at risk may indeed contribute to the willingness to try new, invasive disease-modifying treatments.

Interestingly, patients indicated that a decline in their functioning level to a certain point was not acceptable. It is, however, generally known that once people reach at this certain point, acceptance levels shift and people tend to adopt a more positive attitude towards their current situation, also known as the ‘response shift’ [25]. This phenomenon was also apparent when comparing the answers of the manifest participants with those of the premanifest participants. Both groups wished for no further deterioration, although the manifest participants already seemed to have accepted their current symptoms.

Participants were less positive about participation in a placebo-controlled trial than in an earlier phase, open-label trial. This phenomenon was also observed in previous studies in ataxia and HD [14, 15]. The authors of these studies suggested that adding an open-label extension study could be helpful to recruit more patients for clinical trials that include placebo arms. In an open-label extension study, participants would be assured that at some point during the study period, they would also receive the study drug. Also, proper explanation of the study design to the participant was suggested to be helpful. However, it is known that the placebo effect in progressive chronic disorders as HD and SCA is large and this should always be taken into account [26].

Participants in this study are willing to travel for genetic interventions and for the expertise of the caregiver. However, it should be noted that our study was conducted in the Netherlands, a relatively small country where travel times rarely exceed four hours. Result could thus be different for patients living in countries and regions in which expert care is more remote.

Most participants mentioned a preference to be treated in an expert center and as early as possible in their disease course. However, preferences regarding other practical and procedural aspects, such as mode of administration, differed between participants. It appears that their preferences are based on personal experiences and thoughts. It will in part be difficult to accommodate these preferences, as some aspects depend for example on the designed and tested route of administration.

One premanifest participant with HD preferably wanted to be treated as late as possible in the premanifest disease stage. The argument for this decision was understandable, however, neuronal cell decline begins long before the first clinical symptoms arise. The TRACK-HD study showed that measurable MRI chances are visible 10 years before the expected disease onset of HD [27]. Furthermore, biomarkers such as neurofilament light chain levels in cerebrospinal fluid are already detec table 20 years before disease onset [28]. From a medical point of view, this means that treatment should be started early in order to prevent or delay clinical manifestation of the disease, although this remains to be proven. Also, it is yet unknown what the optimal time point in the premanifest phase would be to start such a genetic intervention, taking risks and complications in account.

One of the limitations of this study is that it comprised a relatively small group of patients. However, saturation was achieved after 10 interviews, and therefore the decision was made not to enroll more participants. Furthermore, the participants who were interviewed were pre-selected by their neurologist. All of them were very willing to contribute to this study and had a relatively high level of background knowledge on this topic. This may have possibly led to bias of the results, for example regarding the willingness to try genetic interventions and to be enrolled in a trial. Therefore, the results of this study may not be generalizable to the entire SCA and HD patient population. Also, the level of functioning of the included participants was rather high); it would be interesting for future research to also include persons who are more severely affected, for example patients who are wheelchair bound or living in a nursing home, to evaluate the effect of the response-shift in these subgroups. For HD, however, this could be difficult given the cognitive defects in laterstages [4].

In general, genetic therapies for brain disorders are still mostly experimental, with some exceptions. When proven effective in symptomatic disease stages, the application in the preclinical stage— to delay or prevent the onset of symptoms— is the obvious next step. Still, the actual implementation of these treatments pose a challenge to the healthcare system, and will come with considerable societal costs and ethical issues such as unequal accessibility.

In conclusion, this qualitative study provides detailed and balanced information that helps healthcare professionals and researchers to understand the patient’s perspective towards genetic interventions, and contributes to the implementation of these new treatments as a form of individualized, patient-centered healthcare for persons with premanifest and manifest SCA and HD. Furthermore, the results may be relevant in the process of inclusion of eligible candidates for trials. Routinely informing premanifest and manifest SCA and HD patients about these future treatments could already be started to take away unrealistic assumptions regarding possible benefits and risks. The fact that these patients appear to be willing to accept high risks of severe side effects in the absence of alternative disease-modifying treatment options, makes them a vulnerable group in need for counselling before starting treatment or participation in a trial.

## Supplementary Material

Supplementary Material

## Data Availability

The data supporting the findings of this study are available on request from the corresponding author. The data are not publicly available due to privacy or ethical restrictions.
