# Supplementary material for: Genetic Interventions for Spinocerebellar Ataxia and Huntington’s Disease: A Qualitative Study of the Patient Perspective
Source: J Huntingtons Dis. 2024 Sep 10;13(3):321–8. doi: 10.3233/JHD-240026 (PMC11492111; doi:10.3233/JHD-240026)
Supplement: Supplementary Material [file jhd-13-jhd240026-s001.pdf]

Supplementary Material

Genetic Interventions for Spinocerebellar Ataxia and Huntington's Disease: A Qualitative Study of the Patient Perspective

Supplementary Material 1. Functioning scales used in Table 1.

1.1. Disease stages of ataxia [16]

Stage	Explanation
0	No gait difficulties
1	Disease onset, as defined by onset of gait difficulties
2	Loss of independent gait, as defined by permanent use of a walking aid or reliance on a supporting arm
3	Confinement to wheelchair, as defined by permanent use of a wheelchair
4	Death

1.2. Functional capacity subscale of the Unified Huntington Disease Rating Scale (UHDRS) [17]

**Occupation**	
0	Unable
1	Marginal work only
2	Reduced capacity for usual job
3	normal
**Finances**	
0	Unable
1	Major assistance
2	Slight assistance
3	Normal
**Domestic chores**	
0	Unable
1	impaired
2	Normal
**Activities of Daily Living**	
0	Total care
1	Gross tasks only
2	Minimal impairment
3	Normal
**Care level**	
0	Full time skilled nursing
1	Home or chronic care
2	Home

1.3. Total functioning capacity score (TFC) of UHDRS

Score	Disease stage
11-13	1
7-10	2
3-6	3
1-2	4
0	5

1.4. Modified Rankin Scale

Score	Explanation
0	No symptoms
1	No significant disability. Able to carry out all usual activities, despite some symptoms
2	Slight disability. Able to look after own affairs without assistance, but unable to carry out all previous activities
3	Moderate disability. Requires some help, but able to walk unassisted
4	Moderately severe disability. Unable to attend to own bodily needs without assistance, and unable to walk unassisted
5	Severe disability. Requires constant nursing care and attention, bedridden, incontinent
6	Death

Supplementary Material 2. Interview guide for semi-structured interviews

Topics

- Current treatment
- Background knowledge about genetic therapies
- Reasons to undergo genetic interventions (i.e., goals, benefits)
- Reasons not to undergo genetic interventions (i.e., risks, side-effects)
- Logistic factors (are these factors of influence in the decision-making process?)
    - Mode and frequency of administration
    - Time investment
    - Location of treatment center and expertise of caregivers
    - Costs
    - Disease stage
- Social factors
    - Opinion of family and friends
    - Opinion of doctors
    - Timing of DNA diagnostics
    - Who should be treated first?
- Participation in trials (what are reasons to participate or to not participate?)

Supplementary Material 3. Data saturation table

Theme	Subtheme	Interview									
		**1**	**2**	**3**	**4**	**5**	**6**	**7**	**8**	**9**	**10**
		**Participant**									
		**4**	**10**	**2**	**3**	**5**	**1**	**7**	**9**	**6**	**8**
Knowledge and assumptions	Background knowledge	+	+	+	+	+	+	+	+	+	+
	Assumptions about benefits	+	+	+	+	+	+	+	+	+	+
	Assumptions about side effects	+	+	+	+	+	+	+	+	+	+
Motives and hopes	Willingness to undergo genetic interventions	+	+	+	+	+	+	+	+	+	+
	Goals	+	+	+	+	+	+	+	+	+	+
	Quality of life	+	+	+	+	-	+	+	+	+	+
	Acceptance of (unknown) risks	+	+	+	+	+	-	+	+	+	+
	Burden in families	-	+	+	+	+	+	+	+	+	+
	Motives for trial participation	+	+	+	+	+	+	+	+	+	+
Preferences	Patient information	+	+	+	+	+	+	+	+	+	+
	Mode of administration	+	+	+	+	+	+	+	+	+	+
	Expertise, location and costs	+	+	+	+	+	+	+	+	+	+
	Other procedural aspects	+	+	+	+	+	+	+	+	+	+
	Timing	+	+	+	+	+	+	+	+	+	+
	Prioritizing	+	+	+	+	+	+	-	+	+	+

Supplementary Material 4. Exemplary quotes from the interviews

Theme	Subtheme	Quote
Knowledge and assumptions	Background knowledge	*P4: 'I have read that two methods are being studied…but it is all a little bit of abracadabra to me.'*
		P3: 'I have chosen not to read about new treatments on patient associations' websites because I find it hard to read sad stories of people who have more problems with the disease than I have.'
	Assumptions about benefits	*P2: 'From what I've read, I expect this treatment would slow down disease progression very much. I would not dare to hope it would cure me'.*
		P10: 'I believe there would be a positive effect of this treatment. I do have faith that the people who work on this for years will develop something that works.'
	Assumptions about side effects	*P8: 'I think side effects would be related to taking down or temporarily shutting down the function of the protein, and I believe this protein is of great importance for the human body'.*
		P3: 'I know there is a chance I could die, but I think I would accept this risk'.
Motives and hopes	Willingness to undergo genetic interventions	*P2: 'The reason for me to want a genetic intervention is that I know how devastating this disease can be for me, so I really want to do something that could change my disease course'.*
		P6: 'If this treatment would become available and I would be eligible, I would be the first person to sign up for it.'
	Goals	*P2: 'I guess maintenance of my current functional status would be too much to ask for such a treatment, but slowing down disease progression would already be very valuable for me.'*
		P9: 'For me it is most important that I would be able to be free of symptoms as long as possible.'
	Quality of life	*P1: 'If this treatment will become available ten years from now, and I will be wheelchair bound with severe ataxia and I am unable to walk and talk, one doesn't need to treat me with injections anymore.'*
		P4: 'If I don't try this treatment and do nothing, I would end up in a wheelchair and I wonder if I would want to be alive at that point.'
	Acceptance of (unknown) risks	*P2: 'If you would ask me the same questions in five years from now, I expect my symptoms to be worse. It is possible that my opinion changes, and that I would be willing to accept more risks than I would do at the moment'.*
		P8: 'For me, uncertainty regarding unknown long-term risks is not as important as the known short-term risks'.

	Burden in families	*P1: 'My mother has lived in a nursing home for 8 years. She was wheelchair bound and could not do anything by herself anymore. Her speech was severely impaired. That is something I would not want for myself'.*
		P10: 'If I were as far in my disease course as my father is now - he lives in a nursing home - I would not want to slow down disease progression anymore.'
		P3: 'If I were further in my disease course, such as my brother who has a higher SARA score and is not able to work anymore, I would be more inclined to try the treatment and accept the risks.'
	Motives for trial participation	*P3: 'If participation in a trial would be risky for me and it only benefited persons I don't know, I wouldn't participate. But if it benefited my own children, I would participate.'*
		P7: 'The main reason to participate in a trial is that I hope the treatment would do something for me. If the scientific community would learn something from that, it would be a nice bonus.'
		P10: 'The big advantage of participation in a trial is that it can be done sooner. If the treatment will be available in 10 or 20 years … it could be too late for me.'
		P4: 'If the drug would be only available in a placebo trial I want to participate because I would still have a 50% chance of receiving the medicine … I would be more inclined to participate in an earlier phase trial with a 100% chance of receiving the medicine.'
Preferences	Patient information	*P2: 'I would want to be informed whether the treatment would be painful or dangerous. I would also want to know the site of the injection in the spinal cord. These are all questions a doctor would be able to answer… I value the doctors' opinion and the experiences the doctor has with the treatment'*
	Mode of administration	*P6: 'If you only have to get the treatment once and after that you're done, that would be most effective and efficient.'*
		P5: 'I would prefer a lumbar puncture. I've had that a few times and in my opinion, it is actually very simple and not that invasive.'
	Expertise, location and costs	*P2: 'I would prefer to go to the expert center for check ups, because the doctors are familiar with the disease and they know other patients with this disease. In my local hospital I would probably have to explain what my disease is.'*
		P1: 'I know that these kind of treatments will only be available in larger medical centers, so I know I have to travel. If they have the expertise, I am willing to travel for that purpose.
		P9: 'For me it is important that I can always contact the same person who knows me, especially because genetic treatment is a process that will take a long time.'

		P10: 'I would not travel abroad for this treatment. If the treatment is good, it will eventually become available in my own country.'
	Other procedural aspects	*P9: 'When I would be further in my disease course, time investment of the procedures would be less burdensome for me because the treatment would then be more necessary.'*
		P1: It's all relative. People with other disorders are in the hospital every week to extend their lives. If I look at it that way, the time investment for this treatment is acceptable.'
		P5: 'My asymptomatic children are in doubt whether they would undergo genetic testing, especially because there is no treatment available. Why would they?'
	Timing	*P8: 'If I were 70 years old and I was living in an institution, unable to function independently, and I received this treatment and still be dependent and wheelchair bound, I am not sure if this treatment would be useful for me.'*
		P5:'I have doubts about premanifest treatment as it can be questionable whether I would develop any symptoms at all; possibly I would have nothing to worry for until I am 80 years old… and then I have had all those lumbar punctures, for what?'
		P9: 'At the moment, I feel completely fine, and taking a risk when I feel so good, that is scary for me…I wouldn't want to be confronted with my diagnosis all the time, because I am not sick yet.'
	Prioritizing	*P1: 'In general, I think healthcare should be accessible for everyone. But if someone is already wheelchair bounded with severe ataxia, delay of progression is possibly not what you want to achieve in that stage. But I think this is a personal decision for each patient.'*
		P4: 'I would say, those who still have any quality of life should be treated first, so they do not deteriorate any further.'
